# Regenerative Approach With Autologous Fat Grafting and Stem Cells for the Treatment of Post‐Traumatic Anal Incontinence: A Case Report

**DOI:** 10.1002/ccr3.71619

**Published:** 2026-01-08

**Authors:** Alexandre da Silva Nishimura, Giuliana Donoso Andrade Silva, Vitor Holmo Figueira, Eduardo Isaac Nishimoto, Pedro Luiz Nishimura Menardi, Igor Rincon Gonçalves Passaglia, Sabrina Thalita dos Reis

**Affiliations:** ^1^ Coloproctologist at Vila Nova Star Hospital, Rede D'Or São Paulo Brazil; ^2^ Radiologist at Ultra One São Paulo Brazil; ^3^ Medical Student at University of São Francisco São Paulo Brazil; ^4^ Plastic Surgeon at Vila Nova Star Hospital, Rede D'Or São Paulo Brazil; ^5^ Laboratory of Medical Investigation (LIM55), Urology Department, Faculty of Medicine University of São Paulo São Paulo Brazil

**Keywords:** anal incontinence, autologous fat grafting, post‐traumatic injury, regenerative medicine, stem cells

## Abstract

Autologous fat grafting enriched with mesenchymal stem cells is a promising minimally invasive regenerative therapy for post‐traumatic anal incontinence. This approach may restore continence and quality of life, even when structural sphincter defects persist.

## 1 Introduction

Fecal incontinence is characterized by the recurrent involuntary loss of liquid or solid feces, and is often associated with stigma and a marked reduction in quality of life [1]. Anal incontinence, which also includes the unintentional release of gas and mucus, has an underestimated prevalence of between 7% and 15% in the general population, with higher rates among the elderly and women [2, 3]. This condition can be classified into three main subtypes: passive incontinence, in which involuntary elimination of feces or gas occurs without the patient realizing it; urge incontinence, characterized by involuntary evacuation even when attempts are made to contain it; and fecal infiltration, which refers to the loss of feces after regular evacuations [3]. Regardless of its severity, it is a relevant clinical challenge, both from a physical and psychological and social point of view, given its morbidity and association with psychopathologies such as depression, anxiety disorders and sexual dysfunction [4, 5].

The pathophysiology of anal incontinence is still not fully understood, but it involves the malfunctioning of anatomical structures that are essential for continence, such as the internal and external anal sphincters, the pelvic floor muscles and the sensorimotor apparatus [6]. Treatment usually begins with conservative measures such as sacral neuromodulation, biofeedback and the use of drugs such as antidiarrheals and laxatives. However, in refractory cases, especially when there are extensive lesions in the internal and external sphincters, surgical approaches are used, including injections of volumizing agents and, in more severe situations, colostomy [7, 8]. In this scenario, regenerative therapies have emerged as promising alternatives, with the aim of restoring sphincter function through tissue regeneration and functional recovery of injured muscle structures.

In recent years, autologous lipografting has gained prominence as a potential regenerative technique, based both on its mechanical volumizing effect and on the high concentration of mesenchymal stem cells derived from the stroma [9]. The use of autologous liposuction provides not only local volume increase, but also benefits such as cell differentiation, angiogenesis and inflammatory modulation. This approach represents a breakthrough in the management of anal incontinence, especially in cases refractory to conventional treatments.

This case report aims to describe a regenerative surgical approach with autologous lipograft for the treatment of post‐traumatic anal incontinence in a patient with a history of complex sphincter injury. It seeks to discuss the clinical, technical and functional aspects involved, as well as the prospect of autologous cell therapy as a minimally invasive therapeutic alternative for the recovery of sphincter function.

## 2 Case History

A previously healthy 17‐year‐old male patient with a history of severe pelvic trauma resulting from a car accident at the age of 15 developed extensive sphincter damage and fecal incontinence. At the time, he underwent a preventive loop colostomy, considering the high risk of irreversible functional loss of the anal canal. In the clinical follow‐up, 2 years after the initial accident, at the age of 17, given the clinical stability and the desire to reverse the intestinal transit, an autologous regenerative approach was started with the aim of recovering anal sphincter function and possible reconstruction of the intestinal transit.

In the preoperative anorectal functional assessment, carried out in October 2022, manometry showed a mean resting pressure of 25.8 mmHg and a mean voluntary contraction pressure of 58.9 mmHg, both reduced values. The anal inhibitory reflex was preserved, with initial rectal sensitivity and minimum rectal capacity within the normal range. The interpretation was internal and external sphincter hypotonia, and training with anal biofeedback was suggested at the time. A new manometry carried out in March 2023 showed a worsening of the parameters, with resting pressure reduced to 14.6 mmHg and voluntary contraction of 48.2 mmHg, while maintaining preserved rectal competence.

## 3 Investigations and Treatment

**FIGURE 1:**
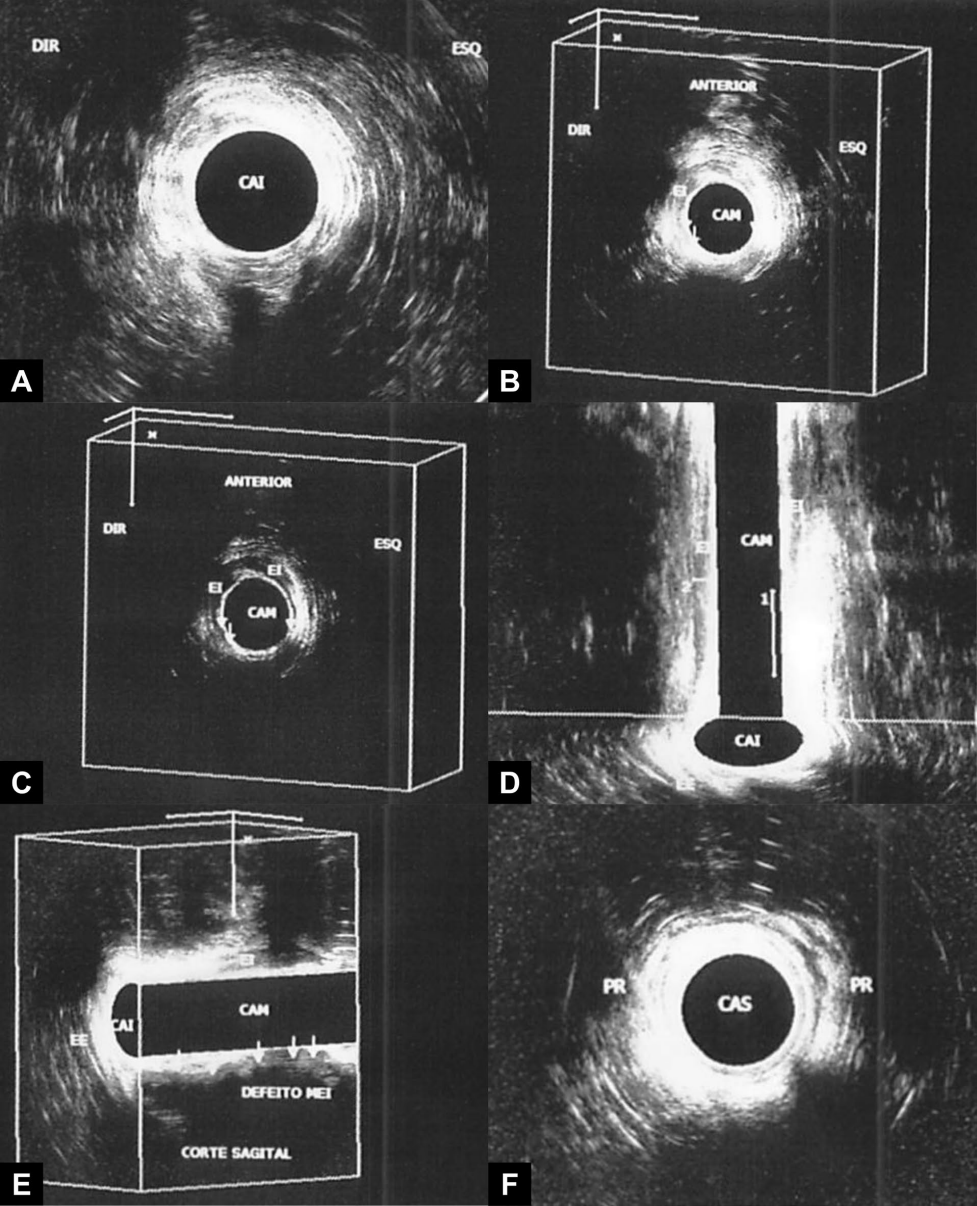
Preoperative 3D endoanal ultrasound showing multiple defects in the internal and external anal sphincters, as well as asymmetry of the puborectalis muscle (Date: 18/01/2024). (A) Upper anal canal: The puborectalis muscle is seen as a hypoechogenic image in the shape of a “V”, with a flaw in the continuity of the muscular cable on the left. (B and C) Middle anal canal: The internal sphincter is seen as a hypoechogenic halo, more evident in the right anterior and anterior quadrants. Two flaws are identified in the other quadrants. The external sphincter muscle also shows interruptions in the right anterolateral and posterior quadrants. (D) Transverse (coronal) section of the three‐dimensional cube: The internal sphincter is visible on the right, with a defect of approximately 14 mm on the left. (E) Sagittal section of the three‐dimensional cube: The internal sphincter is absent in the posterior quadrant, as well as a defect in the external sphincter. (F) Inferior anal canal: The external sphincter muscle shows defects in the right anterolateral and posterior quadrants.

A three‐dimensional endoanal ultrasound was performed in January 2024, which showed failure of the puborectalis muscle cable on the left, as well as failures in multiple quadrants of the internal sphincter in the middle anal canal (Figure 1). In the middle anal canal, the internal sphincter showed a more visible defect in the right anterior quadrant, while the external sphincter showed defects in the right and posterior anterolateral quadrants. In the coronal section of the three‐dimensional cube, a defect of approximately 14 mm was seen to the right of the internal sphincter. These structural alterations supported the hypothesis of extensive post‐traumatic sphincter injury.

Considering the degree of functional and anatomical compromise, a regenerative technique with autologous perianal fat grafting enriched with mesenchymal stem cells (MSCs) was indicated, with the goal of promoting strengthening and potential regeneration of the anal sphincter complex.

### 3.1 Preparation of the Graft

The approach followed an established protocol based on autologous lipograft. Initially, Klein's solution was infiltrated into both iliac fossae (250 mL per side), consisting of saline, adrenaline, ropivacaine and lidocaine. The aim of the infiltration is local vasoconstriction, analgesia and facilitation of liposuction, as well as minimizing bleeding and tissue trauma. After infiltration, a latency period of approximately 10 min was waited, allowing the solution to have a complete vasoconstrictive and anesthetic effect on the infiltrated areas.

Subsequently, liposuction of the subcutaneous adipose tissue was carried out in the previously infiltrated areas, using 3–4 mm cannulas coupled to a controlled negative pressure system, with the aim of extracting viable fat for regenerative purposes. The collected material was subjected to spontaneous decantation and emulsification using 10 mL syringes coupled to an emulsifier, separating the intermediate fraction rich in adipocytes and MSCs.

This process allows the content to be separated into three phases: the oily supernatant, the aqueous component (residual Klein's solution and red blood cells) and the intermediate fraction rich in viable adipocytes and stem cells. The intermediate portion, rich in adipocytes and stem cells, was subjected to a cell isolation process, discarding the oily supernatant and Klein's solutions, with the aim of concentrating mesenchymal stem cells or pluripotent cells. The technique may involve washing with sterile saline solution, filtering and occasional gentle centrifugation, while respecting cell viability. In the meantime, we emulsify the fat using two 10 mL syringes connected to a fat emulsifier. The isolated cells are then reincorporated into the purified adipose tissue, forming a highly regenerative biological compound with the potential for differentiation and integration into sphincter muscle tissue.

### 3.2 Treatment

**FIGURE 2:**
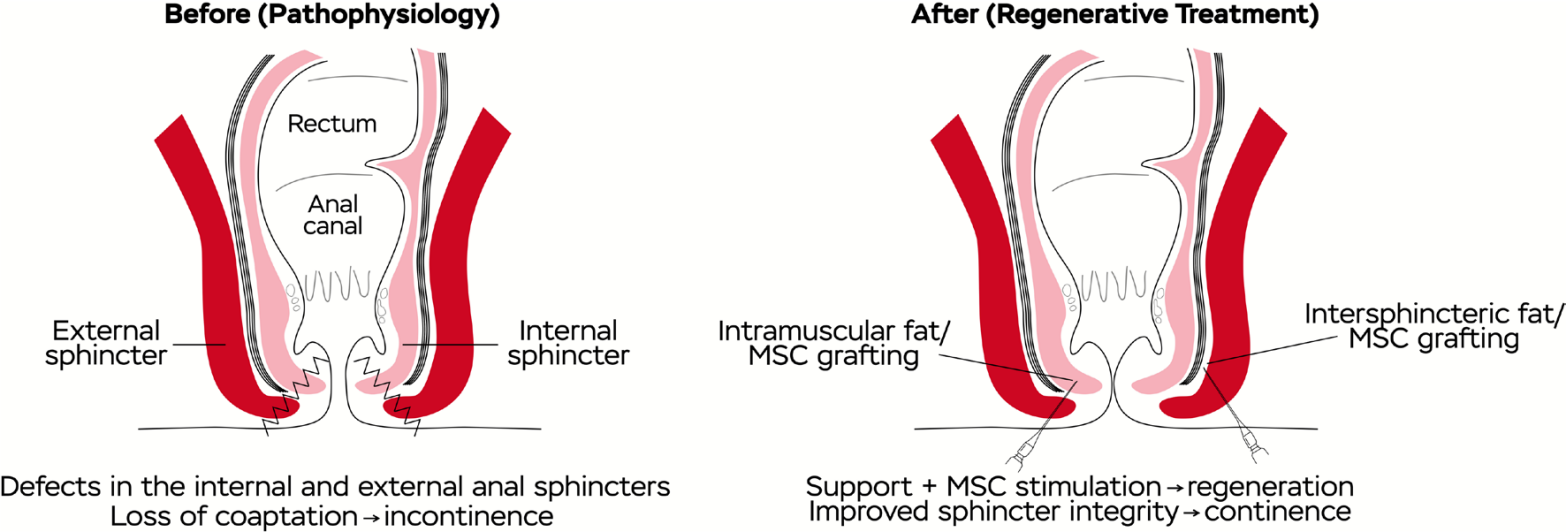
Schematic diagram of sphincter injury and regenerative treatment.

The regenerative mixture was applied to the anal sphincter complex under intraoperative ultrasound guidance, using fine cannulas and taking care to respect the functional anatomy while avoiding additional trauma to the injured area. The injection sites were defined based on preoperative imaging, physical examination, and three‐dimensional endoanal ultrasonography findings. In practical terms, the procedure involved a dual‐plane approach: (i) intramuscular injections were delivered directly into the fibers of the internal anal sphincter, aiming to promote cellular integration and tissue regeneration within the muscular layer; and (ii) injections within the intersphincteric plane, located between the internal and external anal sphincters, provided a biological scaffold to reinforce and stabilize the damaged sphincteric structures. This combined strategy was designed to offer both volumetric support and biological stimulation, thereby enhancing the regenerative response and facilitating functional recovery of the anal sphincter complex. Figure 2 provides a schematic representation of the anal sphincter anatomy, the injury process, and the stem cell–based therapeutic approach.

During the application, several technical precautions were emphasized to prevent iatrogenic injury. Particular care was taken to preserve the pudendal nerve and its terminal branches, which play a critical role in both motor control of the external anal sphincter and sensory function of the anal canal. In addition, attention was directed to the superior, middle, and inferior hemorrhoidal vessels, as inadvertent puncture could lead to significant bleeding or hematoma formation. The anal and rectal mucosa were meticulously preserved to prevent ischemia, necrosis, or fistula formation. Notably, mucosal perforation represents the main intraoperative warning sign, as it may result in the iatrogenic development of an anorectal fistula and compromise the therapeutic outcome. Continuous intraoperative endoanal ultrasound guidance was therefore employed to ensure accurate graft deposition, minimize the risk of inadvertent structural injury, and enhance procedural safety.

This approach aims to promote tissue regeneration and strengthen the sphincter muscles, using autologous material, with a low risk of immunological rejection and without the need for synthetic grafts.

## 4 Outcome and Follow‐Up

**FIGURE 3:**
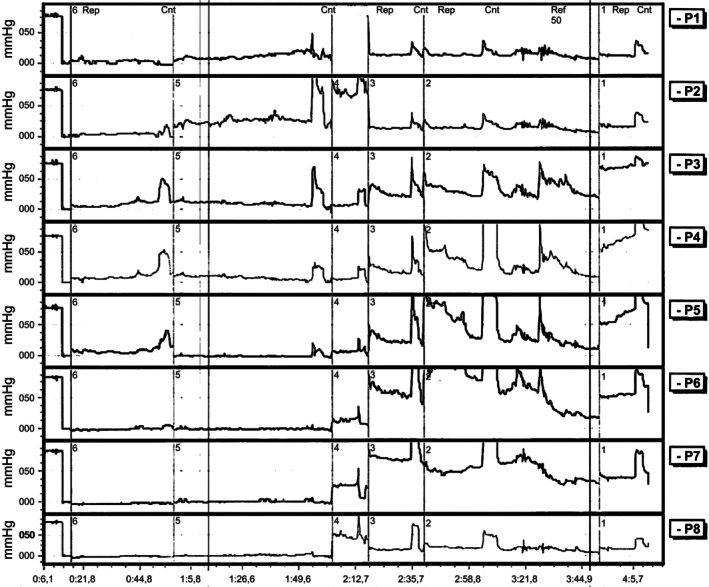
Postoperative anorectal manometry tracing showing resting and exertional hypotonia (Date: 17/10/2024).

After the lipograft, the clinical evolution was satisfactory. In October 2024, a new anorectal manometry showed a significant improvement in blood pressure parameters, with a mean resting pressure of 38 mmHg and a mean stress pressure of 101 mmHg, suggesting an increase in sphincter tone. Although the voluntary striated muscles were still hypotonic and manometry showed 58% asymmetry, a functional anal canal of 4 cm was observed with a preserved rectoanal reflex. Rectal capacity was reduced (maximum tolerated volume: 80 mL), probably due to shunt proctitis (Figure 3).

Subsequent three‐dimensional endoanal ultrasound maintained the structural findings of internal and external sphincter failures and puborectalis muscle asymmetry, with no significant changes after fat injection. Even so, given the functional improvement demonstrated in the physiological tests and the absence of symptoms, reconstruction of the intestinal transit was indicated and performed approximately 8 months after the lipograft.

The colostomy was reversed around 8 months after the lipograft, with excellent clinical progress. Currently, the patient maintains full continence for gas, liquid and solid feces, with recovery of sphincter function and return to normal activities without limitations. There have been no post‐operative complications or need for further interventions to date.

## 5 Discussion

Fecal incontinence represents a significant clinical challenge, profoundly impacting patients' quality of life. As described in this report, the condition can arise as a sequela of complex pelvic trauma, resulting in extensive sphincter lesions and severe functional loss [1, 2]. The case presented illustrates the complexity of managing this post‐traumatic condition in a young patient, where conventional therapeutic options may be limited or insufficient to restore continence satisfactorily [7, 8].

The initial therapeutic approach for FI usually involves conservative measures, such as dietary modifications, physiotherapy with biofeedback and the use of medication. However, in refractory cases or those associated with significant anatomical sphincter defects, such as that observed in this patient after evaluation by anorectal manometry and three‐dimensional endoanal ultrasound, more invasive interventions become necessary [6, 7]. Endoanal ultrasound was crucial in identifying structural flaws in the internal and external sphincters, as well as in the puborectalis muscle, corroborating the traumatic etiology of incontinence and guiding the therapeutic strategy.

In this context, autologous fat grafting has emerged as a promising alternative, exploiting both the volumizing effect of the fat graft and the regenerative potential of the adipose tissue‐derived mesenchymal stem cells (ADSCs) present in the vascular stroma [9]. Recent literature has corroborated the efficacy and safety of this technique in the treatment of FI. A study by Jeong et al. [9] evaluated 35 patients (adults) with fecal incontinence treated with autologous lipograft, reporting symptomatic improvement in 82.9% of cases, with no serious complications, concluding that it is an effective, safe and low‐cost alternative. Similarly, Pinto et al. [10] demonstrated the successful application of ultrasound‐guided anal fat grafting in children with FI secondary to anorectal malformations, observing stable improvement in bowel function and quality of life, associated with an increase in the thickness of the sphincter apparatus on follow‐up ultrasound.

The present case is in line with these findings, demonstrating a significant functional improvement after perianal autologous lipograft, evidenced by a significant increase in resting and stress pressures on control anorectal manometry. The mean resting pressure increased from 14.6 to 38 mmHg, and the mean exertional pressure rose from 48.2 to 101 mmHg. This functional improvement, which culminated in the successful reconstruction of intestinal transit and the recovery of full continence for gas and feces, suggests that lipografting can promote not only a filling effect, but also possible functional modulation or tissue regeneration, even if the structural changes on ultrasound were not significant at follow‐up. The discrepancy between the manometric functional improvement and the absence of significant sonographic changes may indicate that the benefits of lipografting go beyond simple macroscopic anatomical restoration, possibly involving neuromodulatory mechanisms or improved local proprioception, as well as the contribution of ADSCs to tissue repair at a cellular level [9].

The protocol used in this case, with enrichment of the liposuction through emulsification to concentrate the fraction rich in adipocytes and MSCs, seeks to enhance the regenerative effect. Although the initial search in the specific literature on MSCs and fecal incontinence did not return direct results with the terms used, the biological rationale for the use of stem cells in muscle lesions is well established, involving cell differentiation, angiogenesis and inflammatory modulation [9]. Intraoperative ultrasound‐guided application allowed precise deposition of the graft in the areas of greatest sphincter impairment, optimizing the therapeutic potential and minimizing risks.

Fecal incontinence can be managed through different surgical approaches, ranging from direct repair of the sphincteric barrier, including sphincter repair, sphincteroplasty, and muscle transposition, to procedures aimed at strengthening the pelvic floor and promoting functional rehabilitation through nerve stimulation [11]. In addition, injectable biomaterial interventions have gained increasing attention since the 1990s, when Shafik conducted the first trials using polytetrafluoroethylene applied to the anal submucosa [11]. Since then, several materials have been investigated to increase anal canal volume and improve continence, including autologous fat grafts, glutaraldehyde cross‐linked collagen, coated carbon beads (such as Durasphere), stabilized hyaluronic acid, and porcine dermal collagen matrix. Early studies using autologous fat transplantation demonstrated promising outcomes, with significant clinical improvement and a reduction in the frequency of incontinence episodes [12, 13, 14].

Among the main advantages of this technique are its low risk of severe complications, technical simplicity, minimally invasive nature, and the possibility of repetition. Moreover, it utilizes the patient's own tissue, thereby minimizing immunological and inflammatory reactions [12, 13, 14]. However, limitations such as uncertainty regarding the long‐term durability of results, variability in fat resorption over time, and the scarcity of long‐term studies still need to be addressed [15, 16]. When compared with other treatment options, such as implantation of an artificial anal sphincter or sacral nerve stimulation, autologous fat grafting is less complex, more accessible, and associated with lower costs. Nevertheless, unlike these approaches, its beneficial effect appears to be mostly short‐term, and robust evidence from randomized clinical trials is still lacking to confirm its efficacy.

Defining the most appropriate timing for regenerative interventions following traumatic injuries of the pelvic floor or sphincter complex is a key determinant of therapeutic success. In general, it is recommended to wait for the resolution of the inflammatory phase and initial wound healing, typically between 3 and 6 months, while elective and regenerative procedures are usually indicated after 6–12 months to reduce the risk of complications such as infection, edema, or tissue fragility [17]. Studies by Malouf et al. and Berkesoglu et al. have shown that delayed interventions, such as secondary sphincteroplasty, yield better results when performed within the first 5 years after trauma, particularly in younger patients, highlighting the existence of a functional window of opportunity [18, 19]. In the present case, the intervention was performed 2 years after the injury, at a time when both the clinical and anatomical conditions were stable, which likely contributed to the favorable functional outcome.

It is important to note the inherent limitations of a single case report. The absence of a control group prevents direct comparisons, and the follow‐up, although it showed excellent clinical results in the short and medium term, requires longer follow‐up to assess the durability of the effect. The decreased rectal capacity observed in postoperative manometry was attributed to shunt proctitis, a factor that can influence the perception of continence and which tends to improve after transit reconstruction. Future prospective, randomized and controlled studies, with a larger number of patients and prolonged follow‐up, are needed to consolidate the role of autologous fat grafting, with or without cell enrichment, in the treatment algorithm for post‐traumatic FI and other etiologies.

This case demonstrates the potential of autologous fat grafting as a minimally invasive and effective approach for the treatment of severe anal incontinence secondary to complex post‐traumatic sphincter injury. The positive functional results, in line with recent literature findings [9, 10], suggest that this regenerative technique can offer a significant improvement in sphincter function and quality of life, representing a valuable therapeutic option, especially in refractory cases or when traditional surgical reconstruction presents challenges.

## Author Contributions

**Alexandre da Silva Nishimura:** conceptualization, data curation, formal analysis, validation, visualization, writing – original draft, writing – review and editing. **Giuliana Donoso Andrade Silva:** formal analysis, investigation, methodology, validation, visualization, writing – original draft, writing – review and editing. **Vitor Holmo Figueira:** formal analysis, investigation, methodology, validation, visualization, writing – original draft, writing – review and editing. **Eduardo Isaac Nishimoto:** formal analysis, investigation, methodology, validation, visualization, writing – original draft, writing – review and editing. **Pedro Luiz Nishimura Menardi:** formal analysis, investigation, methodology, validation, visualization, writing – original draft, writing – review and editing. **Igor Rincon Gonçalves Passaglia:** formal analysis, investigation, methodology, validation, visualization, writing – original draft, writing – review and editing. **Sabrina Thalita dos Reis:** conceptualization, formal analysis, investigation, methodology, validation, visualization, writing – original draft, writing – review and editing.

## Funding

The authors have nothing to report.

## Ethics Statement

This case report was approved by the Research Ethics Committee of the Hospital das Clínicas, Faculty of Medicine, University of São Paulo (CAAE: 90764925.8.0000.0068).

## Consent

Written informed consent was obtained from the patient for the publication of this case report.

## Conflicts of Interest

The authors declare no conflicts of interest.

## Data Availability

The data that support the findings of this study are available from the corresponding author upon reasonable request.
